# EBV-Associated Cancer and Autoimmunity: Searching for Therapies

**DOI:** 10.3390/vaccines3010074

**Published:** 2015-02-05

**Authors:** Giovanni Capone, Candida Fasano, Guglielmo Lucchese, Michele Calabrò, Darja Kanduc

**Affiliations:** 1 Department of Biosciences, Biotechnologies and Biopharmaceutics, University of Bari, Bari 70126, Italy; E-Mails: g.capone@biologia.uniba.it (G.C.); candida.fasano@gmail.com (C.F.); michele.calabro@hotmail.it (M.C.); 2 Brain and Language Laboratory, Free University of Berlin, 14195 Berlin, Germany; E-Mail: guglielmo.lucchese@hotmail.com

**Keywords:** EBV EBNA1, cancer, autoimmunity, peptide matching, low-similarity peptides, anti-EBV vaccine, peptide-therapy

## Abstract

Epstein-Barr virus (EBV) infects B-, T-, and NK cells and has been associated not only with a wide range of lymphoid malignancies but also with autoimmune diseases such as lupus erythematosus, rheumatoid arthritis and, in particular, multiple sclerosis. Hence, effective immunotherapeutic approaches to eradicate EBV infection might overthrow cancer and autoimmunity incidence. However, currently no effective anti-EBV immunotherapy is available. Here we use the concept that protein immunogenicity is allocated in rare peptide sequences and search the Epstein-Barr nuclear antigen 1 (EBNA1) sequence for peptides unique to the viral protein and absent in the human host. We report on a set of unique EBV EBNA1 peptides that might be used in designing peptide-based therapies able to specifically hitting the virus or neutralizing pathogenic autoantibodies.

## 1. Introduction

Epstein-Barr virus (EBV) is implicated in the development of a wide range of lymphoproliferative disorders, including Burkitt’s lymphoma [1], classic Hodgkin’s lymphoma (HL) [2], non-Hodgkin lymphoma (NHL) [3,4], nasal NK-cell lymphoma [5,6] and, in addition, in nasopharyngeal carcinoma and a subset of gastric cancers [7,8,9].

Moreover, autoimmune diseases may follow EBV-infection. In fact, a potential role of EBV in systemic lupus erythematosus (SLE) has been suggested [10], and development of lupus-like autoantibodies (AAbs) following immunization with Epstein-Barr nuclear antigen 1 (EBNA1) fragments has been reported in animals [11]; links between rheumatoid arthritis (RA) and EBV have been reported [12,13]; also, association between anti-EBNA titers and risk of multiple sclerosis (MS) has been described [14] and oligoclonal bands immunoreactive with EBV EBNA1 have been found in patients with MS [15].

On the whole, given the fact that EBV has been detected in all populations and geographical areas [16] and that the virus has an efficacious immune escape strategy [17], EBV-infection may reasonably be considered as contributing to the continuously increasing incidence of cancer and autoimmunity worldwide. Indeed, for example, HL and NHL have been ranked respectively as the 25th and 10th most common cancers worldwide in 2012 [18]. 

Likewise, MS cases increased in the period 2008–2013 (from 2.1 to 2.3 million) [19]; the incidence of SLE is nearly tripled in the last 40 years of the 20th century [20], and RA affects approximately 1% of the worldwide population [21]. Hence, anti-EBV immunotherapies might overthrow the incidence of highly common cancers such as lymphomas as well as the increasing incidence of autoimmune diseases such as SLE and MS. However, notwithstanding the need of fighting EBV infection, currently effective vaccines are not yet available.

Here, we use the concept that immunogenic properties are allocated in rare peptide modules along a protein sequence [22,23,24,25] and search the EBV EBNA1 sequence for peptides unique to the viral protein and absent in the human host. We find and describe a set of unique EBV EBNA1 peptides that might be used in designing peptide-based vaccines able to specifically hit the viral protein without crossreacting with the host proteins. The present data appear of special interest since might lead to vaccination protocols for a global EBV eradication. Moreover, such unique EBV epitopic peptides might find application to specifically neutralize pathogenic AAbs associated with the autoimmune diseases that have been related to EBV infection.

## 2. Experimental Section

Analyses were conducted on the primary amino acid (aa) sequence of EBV EBNA1 protein (UniProtKB/Swiss-Prot ID: Q3KSS4, 641 aa) from EBV strain GD1, GenBank: AY961628.3 (http://www.ebi.ac.uk/ena/data/view/AY961628) [26]. As a control, EBV GP350 protein (UniProtKB/Swiss-Prot ID: Q3KST4, 856 aa) was also analyzed.

Sequence similarity analysis of the EBNA1 protein *vs.* the human proteome was performed using pentapeptides as scanning probes. The viral protein was dissected into 5-mers sequentially overlapping by four residues (*i.e.*, MSDEG, SDEGP, DEGPG, EGPGT, *etc.*). For each viral 5-mer, the human proteome was searched for instances of the same identical 5-mer using PIR match program (pir.georgetown.edu/pirwww/search/peptide.shtml) [27]. Any such occurrence was termed a match.

The cross-reactivity potential for each pentapeptide sharing was evaluated using Immune Epitope Database and Analysis Resources (IEDB; http://www.iedb.org/) [28] to search for EBNA1-derived B- and/or T-cell epitopes that had been experimentally validated as immunopositive in the human host.

Consensus peptide sequences were defined by ClustalW multialignment analysis (http://www.uniprot.org/align/) [29] of three EBV EBNA1 sequences corresponding to: Q3KSS4 (from GD1 strain, NCBI Tax ID: 10376); P03211 (from B95-8 strain, NCBI Tax ID: 10377); and Q1HVF7 (from AG876 strain, NCBI Tax ID: 82830).

## 3. Results

### 3.1. Peptide Commonality between EBNA1 and the Human Proteome

As exhaustively discussed by Benjamin *et al.* [30], immune “determinants are conformational in the sense that the antibody combining sites will bind with a measurable affinity only to that population of antigen conformers which presents a complementary constellation of interacting side chains. It follows that antigenic determinants are topographic, *i.e.*, composed of structures on the protein surface. Topographic determinants may be contained within a single segment of the amino acid sequence (but not necessarily involving all contiguous residues in the segment), or assembled from residues far apart in the amino acid sequence but brought together on the surface by the folding of the protein antigen” [30].

By considering that conformational and sequential determinants do not imply different antigenic binding mechanisms [30], and that: (1) specific Abs can be raised against peptides of undefined conformation [31]; (2) short synthetic peptides capable of eliciting protein-reactive sera are frequently represented in the primary sequence of a protein [32]; (3) such immunogenic peptides are frequently represented in the primary sequence of a protein [33]; and (4) generation of protein-reactive Abs by short peptides is an event of high frequency [34], research on antigen epitopes has been mainly directed toward linear aa sequences as documented by epitope databases such as IEDB [28], Tri-peptide similarity and Propensity scores (SVMTriP) [35], Linear Epitope Prediction by Propensities and Support Vector Machine (LEPS) [36], and Linear B-cell Epitope Prediction Server (LBtope) [37].

Here, analyses focused on short determinants formed by sequential contiguous aa and used a measurement unit defined by peptide length (*i.e.*, the pentapeptide) for peptide uniqueness comparisons. In fact, a number of reports have reiterated the concept that a grouping of 5 aa residues can be immunogenic [38,39,40,41,42,43,44] and have immunorecognition capability [44,45,46,47,48]. Hence, a pentapeptide may represent an appropriate length unit to be used for analyzing the potential for immune crossreactivity of peptide sharing among proteins [24,49,50].

EBV EBNA1 antigen was chosen since it is constitutively expressed in EBV malignancies. In fact, it is often the only latent EBV antigen expressed in Burkitt’s lymphoma and nasopharyngeal carcinoma [51]; it appears to be involved in the development of gastric cancer [52]. Moreover EBNA1 represents a prime target for T-cell-based immunotherapy [53,54,55].

**Figure 1:**
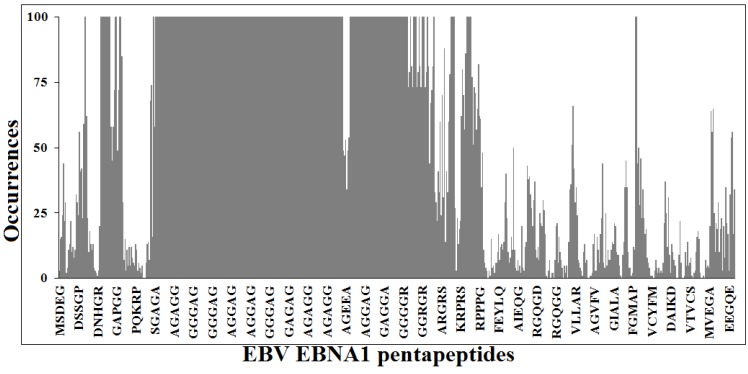
Pentapeptide identity platform shared between Epstein-Barr virus (EBV) Epstein-Barr nuclear antigen 1 (EBNA1) protein and the human proteome. Peptide aa sequences in one letter code.

Figure 1 illustrates the similarity profile of EBV EBNA1 protein sequence to the human proteome at the pentapeptide level. It can be seen that the most part of the 637 pentapeptides that sequentially form the viral protein, are repeatedly present in human proteins. Namely, 622 out of 637 viral pentapeptides are present 66,052 times (including multiple occurrences) in 7312 human proteins [56].

Qualitatively, analysis of the pentapeptide overlap using PIR database highlights that (1) human proteins playing crucial roles in cell functions are involved in the peptide sharing and, in addition; (2) in many instances, many consecutively overlapping pentapeptides forming long peptide stretches are shared. For example:

(1) The EBNA1_54–62_PGAPGGSGS nonapeptide is common to the human protein zyxin (UniProtKB/Swiss-Prot IDs: Q15942, ZYX_HUMAN), a component of a signal transduction pathway that mediates adhesion-stimulated changes in gene expression [57];
(2) The AGAGGAGAG nonapeptide is repeated five times in EBNA1, and is shared with the human ubiquitin-conjugating enzyme E2 Q1 (UniProtKB/Swiss-Prot IDs: Q7Z7E8, UB2Q1_HUMAN) that catalyzes the covalent attachment of ubiquitin to other proteins [58];
(3) The GGGAGGAGG nonapeptide is repeated five times in EBNA1, and is shared with the transcription factor jun-B (UniProtKB/Swiss-Prot IDs: P17275, JUNB_HUMAN). JUNB is a transcription factor involved in regulating gene activity following the primary growth factor response [59] and inhibits proliferation and transformation in B-lymphoid cells [60];
(4) In addition, the GGGAGGAGG nonapeptide is also present in the human far upstream element-binding protein 2 (UniProtKB/Swiss-Prot IDs: Q92945, FUBP2_HUMAN). FUBP2 binds to the dendritic targeting element and may play a role in mRNA trafficking. FUBP2 also mediates exon inclusion in transcripts that are subject to tissue-specific alternative splicing [61];
(5) The EBNA1_40–50_GRGRGRGRGRGRGRG undecapeptide is also present in the human small nuclear ribonucleoprotein SmD1, a core component of the spliceosomal U1, U2, U4 and U5 small nuclear ribonucleoproteins (snRNPs), the building blocks of the spliceosome [62]. Importantly, this dipeptide Gly-Arg repeat crossreacts with Abs against an SmD-like epitope recognized by sera from SLE patients [63], thus possibly underlying the EBV-SLE association [10,11].

Moreover, it has to be underlined that the above listed peptide identities occur along the central 200 aa long Gly-Ala repeat of EBNA1, a viral region that has immunosuppressive properties since it may affect MHC I restricted responses by inhibiting antigen processing via the ubiquitin/proteasome pathway [64,65].

### 3.2. Searching for a Vaccine: Unique EBNA1 Sequences

Translating the data exposed above to the context of the present study, Figure 1 illustrates the concept that using vaccines based on the entire EBNA1 antigen might potentially lead to a plethora of crossreactions with proteins exerting fundamental roles in the human host. On the contrary [22,23,24,25], only pentapeptides unique to EBNA1 might lead to peptide-based vaccines exempt of crossreaction. The 15 viral pentapeptides absent in the human proteome are illustrated in Table 1.

**Table 1:** Peptide profile of EBV EBNA1 protein primary sequence *vs.* the human proteome: the unique EBNA1 identity spots at the pentapeptide level.

Position ^a^	Sequence ^b,c^	Position ^a^	Sequence ^b,c^	Position ^a^	Sequence ^b,c^
80–84	**IGCKG**	467–471	KHRGQ	584–588	MTKPA
81–85	**GCKGA**	476–480	PKFEN	588–592	**APTCN**
82–86	**CKGAH**	499–503	**EEGNW**	589–593	**PTCNI**
461–465	KGGWF	500–504	**EGNWV**	598–602	CSFDD
464–468	WFGKH	561–565	YFMVF	609–613	WFPPM

The EBV EBNA1 pentapeptides with zero similarity to the human proteome (e.g., viral pentapeptides absent in the human proteins) are sequentially listed by aa position along the viral protein. ^a^ Aa position along the viral protein; ^b^ Aa sequences given in one-letter code; ^c^ Consecutively overlapping pentapeptides given in bold.

The pentapeptides described in Table 1 might effectively be a basis for efficacious anti-EBNA1 vaccines also in light of the fact that such unique viral peptide sequences are part of EBNA1 epitopes already experimentally validated as immunopositive in the human host. Indeed, as shown in Table 2, 26 EBNA1-derived epitopes cataloged at IEDB and validated as immunopositive in humans host 13 out of the 15 unique viral pentapeptides described in Table 1.

Sequence comparison analysis also shows that 6 out of the 15 pentapeptides unique to EBNA1 from EBV strain GD1 are also conserved in EBNA1 from B95-8 and AG876 strains (see Table 3). This means that using peptide sequences described in Table 3 might offer the possibility of hitting different EBV strains using a single vaccine preparation.

**Table 2:** Thirteen out of the 15 pentapeptides unique to EBV EBNA1 and absent in the human proteome, are distributed among 26 EBV EBNA1-derived epitopes are immunoreactive in humoral and/or cellular immunoassays.

IEDB ID ^a^	Epitope Sequence ^b,c^	Immune Context	References
1219	aevlkdaikdlvMTKPAptc	B	[66]
8395	dggrrkKGGWFGKHr	T	[67,68]
8397	dggrrkKGGWFgrhr	T	[69]
11651	EEGNWVagvfvyggsktslynlrrg	T	[53]
26761	ikdlvMTKPAPTCNI	T	[70]
30951	KGGWFGKHRGQggs	B,T	[71,72]
39079	lresivcYFMVFlqthifae	T	[67]
39080	lresivcYFMVFlqthifaevlkda	T	[53]
45378	nPKFENiaeglrall	T	[67,68,69]
45379	nPKFENiaeglrallarshv	T	[55,73]
45380	nPKFENiaeglrallarshverttde	T	[74,75]
48948	ppWFPPMvegaaa	T	[76]
49056	pqpgplresivcYFMVFlqt	T	[53]
49593	PTCNIkatvCSFDDgvdlpp	T	[67,69]
49594	PTCNIkvtvCSFDDgvdlppWFPPM	T	[53]
55299	rpqkrpscIGCKGthggtga	B	[66]
55336	rpscIGCKGthggtg	T	[77]
55684	rrpqkrpscIGCKGt	T	[67,69]
56433	rvtvCSFDDgvdlppWFPPM	T	[67]
59875	snPKFENiaeglrvllarsh	T	[54,55]
67891	vcYFMVFlqthifae	T	[70]
69559	vlkdaikdlvMTKPAPTCNI	T	[67,69]
73861	YFMVFlqthifae	T	[76]
73862	YFMVFlqthifaevl	T	[77]
93570	PKFENiaeglr	T	[78]
118828	gsgprhrdgvrrpqkrpscIGCKGthggtg	B	[79]

^a^ EBV EBNA1-derived epitopes are listed according to increasing IEDB ID number. For further details and reference(s) see IEDB [28]; ^b^ Only EBV EBNA1-derived epitopes that had been experimentally validated as immunopositive in the human host are reported; ^c^ In each epitope, EBV EBNA1 pentapeptide(s) absent in the human proteome are given in capital.

**Table 3:** Conservation of EBNA1 unique peptide regions among EBV GD1, B95-8, and AG876 strains.

EBV Strain	ID	Consensus Peptide Sequences				
GD1	Q3KSS4	IGCKG	GKHRG	APTCNI	CSFDD	WFPPM
B95-8	P03211	IGCKG	GKHRG	APTCNI	CSFDD	WFPPM
AG876	Q1HVF7	IGCKG	GKHRG	APTCNI	CSFDD	WFPPM

EBNA1 sequences were aligned using ClustalW program (http://www.uniprot.org/align/) [29]. The analyzed sequences are reported by SwissProt/UniProtKB ID. EBV strains are described at www.uniprot.org.

## 4. Discussion

Numerous immunological approaches have been explored to fight EBV. A few examples are:

- EBNA1 targeting to dendritic cells to stimulate protective T-cell responses [80];
- EBV-specific cytotoxic T-lymphocytes to control EBV-related lymphoproliferation [81];
- a live recombinant virus, expressing under the 11K vaccinia promoter the major EBV membrane antigen BNLF-1 MA (GP 220-340) to protect against and/or delay EBV infection [82];
- GP350(1-470)-based vaccines in order to prevent the virus binding to CD21 on B-cells [83,84,85];
- EBV vaccines based on virus-like particles that mimic the structure of the parental virus but lack the viral genome [86];
- adoptive transfer of EBV specific CD8^+^ T cell clones [87,88].

However, in spite of the numerous and intensive studies, currently there is no specific treatment/vaccine against EBV infection [89].

**Figure A1:**
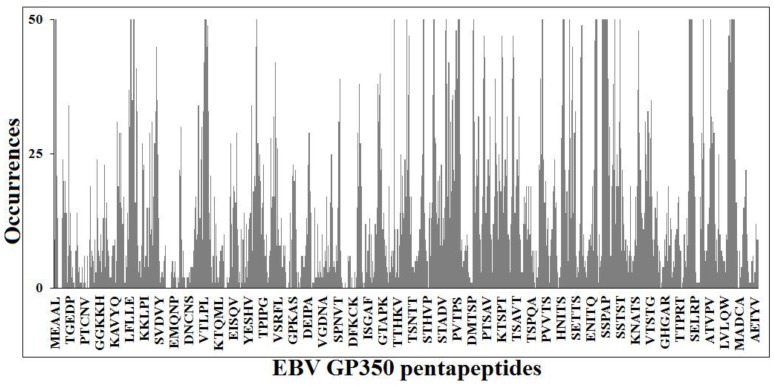
Pentapeptide identity platform shared between EBV GP350 protein and the human proteome. Peptide aa sequences in one letter code.

The present study proposes the principle of peptide uniqueness [22,23,24,25] to construct and develop specific and efficacious EBV vaccines that are exempt from potential crossreactions. In this regard, our findings might also help avoid potential crossreactions in EBV GP350 antigen-based vaccine currently under trial to prevent infectious mononucleosis [83,84]. As a matter of fact, the pentapeptide identity platform shared by EBV GP350 antigen and the human proteome (Appendix Figure A1) reproposes a relevant pentapeptide overlap between EBV GP350 and human proteins, in analogy to the results obtained for EBV EBNA1 and illustrated in Figure 1.

A lack of crossreactivity acquires a clinical importance also in light of the fact that autoimmunity has been associated to high anti-EBV immune responses in the human host. Indeed, increased anti-EBV EBNA1 immune responses predict conversion to MS [90,91], and, likewise, high immune responses to EBV have been found in individuals with systemic and organ specific autoimmune disorders such as RA and SLE [92]. In particular, it is of special relevance to the present study that SLE patients are characterized by a heterogeneous immune response to a dipeptide repeat GR (Gly-Arg). As reported above, such a dipeptide repeat GR represents a shared sequence between EBNA1 and SmD1 and is also a well characterized epitope (IEDB ID: 117518) [64,93].

Such an approach would have the added advantage of preventing crossreactions that appear to be at the basis of the autoimmune diseases presumably associated with immune responses that follow EBV infection. Given the theoretically highest safety of vaccines based on peptides unique to infectious pathogens, intensive prophylactic campaigns of anti-EBV vaccination might be possible, thus promising a global eradication of EBV in the human population.

Therapeutically, our findings open the way to verifying the possibility of using unique EBV epitopic peptides to treat autoimmune diseases related to EBV infection. In fact, short peptides are too small to stimulate antigenic responses to pathogenic regions of autoantigens and may represent effective tolerogens capable of anergizing autoreactive T cells [94,95]. Therefore, unique EBV epitopic peptides might be used to selectively block and neutralize circulating autoreactive AAbs in EBV-associated autoimmune diseases. In this regard, we already used the concept of peptide uniqueness to search *Pemphigus vulgaris* (PV) autoantigen desmoglein-3 (Dsg3) for peptide sequence(s) to be used for blocking autoreactive AAbs [96,97]. The search led to Dsg3_49–60_REWVKFAKPCRE peptide sequence that (1) is uniquely expressed in Dsg3 and, consequently, cannot evoke collateral secondary autoimmune cross-reactions; (2) is allocated in a Dsg3 domain involved in the intramolecular epitope spreading characterizing the progression of PV from mucous to muco-cutaneous stage [98]; (3) did not produce pathogenic Abs in an animal model [99]. Remarkably, topical administration of the Dsg3_49–60_REWVKFAKPCRE peptide was able to stably reverse a terminal stage of PV complicated by diabetes and cataract disease [100].

## 5. Conclusions

The present study applies the concept of peptide uniqueness to develop new therapeutic approaches against EBV infection and the associated cancer and immune pathologies. The data warrant further studies and research since treatments based on peptides uniquely owned by EBV would offer high specificity as well as the advantage of a lack of adverse events in the host.
